# Cognitive characteristics in Chinese non-demented PD patients based on gender difference

**DOI:** 10.1186/s40035-018-0120-1

**Published:** 2018-07-19

**Authors:** Ke Yang, Bo Shen, Da-ke Li, Ying Wang, Jue Zhao, Jian Zhao, Wen-Bo Yu, Zhen-yang Liu, Yi-lin Tang, Feng-tao Liu, Huan Yu, Jian Wang, Qi-hao Guo, Jian-jun Wu

**Affiliations:** 1 Department of Neurology and Institute of Neurology, Huashan Hospital, Fudan University, 12 Wulumuqi Zhong Road, Shanghai, 200040 China; 2 Department of Neurology, Jing’an District Center Hospital of Shanghai, 259 Xikang Road, Shanghai, 20040 China

**Keywords:** Parkinson’s disease, Cognition, Gender effect, Cognitive deficits

## Abstract

**Background:**

Cognitive impairment is one of the non-motor symptoms in Parkinson’s disease (PD). In the present study, we aim to examine the cognitive function of non-demented Parkinson’s disease patients and compare the results between male and female patients as well as control groups in search of any gender effect.

**Methods:**

Sixty PD Patients (30 males and 30 females) from the Movement Disorders Clinic at Huashan Hospital Affiliated to Fudan University were recruited to participate in the study. One hundred age and gender matched control subjects without neurological or psychiatric disorders were voluntarily recruited. The participants were administered measures of cognition in five domains including memory, language, spatial processing abilities, attention and executive function.

**Results:**

PD patients attained significantly lower scores in the visual spatial function, language and attention/executive function compared with the control group. Anti-parkinsonian treated patients performed worse in Rey-copy score, Clock Drawing Test (CDT) and Verbal Fluency-City than untreated ones. In regard to gender differences, though no general cognitive differences were found in Mini-mental State Examination (MMSE), men surpassed women on Boston naming test (BNT) while women were superior on Auditory Verbal Learning Test-long (AVLT) delayed cued recall test.

**Conclusions:**

Cognitive impairments were common in PD patients even in the absence of dementia. PD patients with anti-parkinsonian medication had worse cognitive impairment than untreated patients. Genders may have different manifestations of cognitive impairment in PD patients.

## Background

Parkinson’s disease (PD) has been considered a debilitating motor disorder, and the non-motor symptoms are gaining more and more attention. Cognitive impairment is a major non-motor symptoms, which greatly influence the quality of life [1]. It is estimated that 25% untreated ‘de novo’ patients have cognitive impairment of varying degrees. Some changes in cognition are subtle thus inconspicuous to the patients and their caregivers [2, 3]. Various studies have been conducted to measure specific cognitive functions in PD patients, such as executive abilities, working memory, visuospatial processing, language and attentional processes [4–6]. However, no agreement has been reached as to a definite neuropsychological profile of non-demented PD patients. Besides, it is reported that more men than women are diagnosed with PD, suggesting a gender difference in PD [7, 8]. Although a few studies addressed the gender differences in PD as well as the influence of estrogen on dopaminergic neurons and related pathways in the brain, most of them adopted general cognitive screening tools such as Mini-mental State Examination (MMSE) or Montreal Cognitive Assessment (MocA), little is known as to the specific cognitive domains influenced by gender [9–12]. Therefore, knowledge about differences in cognition between men and women with PD and about the pathophysiology underlying those differences may enhance the accuracy and effectiveness of clinical assessment and treatment of the disease.

The current study examined the five domains of cognitive function in non-demented PD patients who were not treated with anticholinergic medications and normal controls, with special emphasis on the comparison between male and female patients. Meanwhile, we performed sub-group analysis regarding medical treatment of PD patients, aiming to eliminate the possible confounding effects of medication and making the groups more comparable. As the effect of anti-parkinsonism medication on cognitive function was complicated and controversial [13–15].

## Methods

### Subjects

Patients were recruited from the Movement Disorders Clinic at Huashan Hospital Affiliated to Fudan University. All patients fulfilled the UK PD Society Brain Bank (PDSBB) diagnostic criteria for PD [16]. A total of 60 PD patients were recruited to participate in the study, including 30 males and 30 females. Every participant underwent a comprehensive neuropsychological assessment as part of a longitudinal study of cognition in PD patients. None of the patients complained of cognitive decline or visual hallucination. Dementia and depression were ruled out according to Diagnostic and Statistical Manual of Mental Disorders IV criteria [17]. Patients with the history of drug or alcohol abuse, cardiovascular disease, insulin - dependent diabetes, head trauma as well as those who underwent surgical relief of PD symptoms were excluded. One hundred age- and gender- matched control subjects with no neurological or psychiatric disorders were voluntarily recruited. The study was approved by the ethics committee of Huashan Hospital and written informed consent was obtained from each subject included in the study after the procedure was fully explained. Demographic and clinical data of all the PD patients are summarized in Table 1.

**Table 1:** Clinical and Demographic Description of PD patients

	PD (*n* = 60)	Male PD (*n* = 30)	Female PD (*n* = 30)
Age(yr)	59.05 ± 9.55	58.67 ± 10.23	59.43 ± 8.98
Age of onset(yr)	54.97 ± 10.42	54.4 ± 11.38	55.53 ± 9.53
Handedness(L/R)(n)	0/60	0/30	0/30
Educational level(yr)	12.86 ± 2.97	13.1 ± 2.94	12.61 ± 3.03
Duration of illness(yr)	4.22 ± 5.33	4.37 ± 4.85	4.07 ± 5.85
Hoehn and Yahr stage	1.73 ± 0.8	1.92 ± 0.81	1.54 ± 0.76
UPDRS—Part III (“off” medication)	24.81 ± 12.04	24.42 ± 13.32	25.19 ± 10.92
Medication(n)	31/60	15/30	16/30
MAOI-B	4	1	3
Dopamine agonist	7	2	5
Levodopa	9	5	4
Levodopa and dopamine agonist	12	7	5
Levodopa and MAOI-B	2	1	1
Dopamine agonist and MAOI-B	1	1	0
Medication(equivalent)	472.74 ± 293.16	483.73 ± 293.69	460.28 ± 302.34
BDI	12.44 ± 9.06	12.48 ± 10.88	12.39 ± 7.08
MMSE	28.98 ± 1.07	28.97 ± 0.81	29 ± 1.29

Note*. UPDRS* Unified parkinson’s disease rating scale, *MAOI-B* Monoamine oxidase inhibitor type B, *BDI* Beck depression inventory, *MMSE* Mini-mental state examination

### Procedure

All the evaluations were conducted or supervised by a licensed clinical neurologist. Stage of illness was determined using the Hoehn and Yahr scale [18]. PD duration was defined as the time between disease onset (self-reported onset of the first cardinal motor manifestation of Parkinsonism, i.e., rest tremor, rigidity, or bradykinesia) and the time of neuropsychological evaluation. The severity of the motor symptoms was assessed using part III of the Unified PD Rating Scale (UPDRS) (examined in the medication “off” phase) [19].

Participants were asked to provide information on their use of medication. Thirty-one of the patients were on anti-parkinsonian treatment at the time of investigation and 29 were untreated. Treatment included MAO-B inhibitors (*n* = 4), L-dopa monotherapy (*n* = 9), dopamine agonist monotherapy (*n* = 7), a combination of L-dopa and dopamine agonist (*n* = 12), a combination of L-dopa and MAO-B inhibitors (*n* = 2), or a combination of dopamine agonist and MAO-B inhibitors (*n* = 1). No patients were asked to change their medication for this study, nor were any receiving psychoactive or anticholinergic medication. Levodopa-equivalent daily dose (LEDD) was calculated according to standard conversion formula [20].

Neuropsychological tests were conducted in the morning under the “on” status, which was 30 to 60 min after taking the anti-parkinsonism medication. Subjects were allowed to take breaks when needed, in order to maximize performances. All tests were conducted according to standard procedure as outlined in test manuals. The test battery, which required approximately 2.0 h to complete, included a screening test of MMSE for global cognitive efficiency [21]. Five cognitive domains were evaluated: memory, language, spatial processing abilities, attention and executive function. All the tests were administered and scored according to published procedures which were shown in Table 2.

**Table 2:** Cognitive Tests

Cognitive domains	Tests	Descriptions
Verbal memory	AVLT [45]	A list of 12 items is presented three times, each followed by free recall testing. After an interference test lasting 5 min, free recall of the list for the fourth time (short delayed free recall). After another 20 min, free and cued recall for the fifth time (long delayed free and cued recall), and choose the right items from a total of 24 (recognition). (Max score of recognition = 24, the rest = 12)
Spatial processing ability	Rey-copy [46]	Copy one complex line-drawing figure without reminding later recall. (Max score = 36)
	CDT [47]	Draw a clock and mark the time 1:50. (Max score = 30)
Non-verbal memory	Rey-delayed recall [46]	20–25 min after copying, recall the complex line-drawing figure. Identify the color of print in which a color name is written rather than the reading of the name itself. (Max score = 110)
Language	BNT [48]	Name 30 line drawings of common objects shown sequentially, each within 20s. (Max score = 30)
	VFT(animals, cities, alternatives) [48]	Name as many animals as possible within 1 min; same for cities and animal-city alternatives.
Attention/executive function	SDMT	Numbers ranging from 1 to 9, with each digit matched to a different geometrical symbol. Write down the digit according to the symbol as quickly as possible.
	TMT [49]	Scan and connect either all numbers (Trail A), or alternating numbers and letters (Trail B), distributed in a spatial array.
	Stroop [50]	Identify the color of print in which a color name is written rather than the reading of the name itself. (Max score = 110)

Note*. AVLT* Auditory verbal learning test, *Rey-copy* Copy of Rey-Osterrieth complex figure, *CDT* Clock drawing test, *Rey-delayed recall* Delayed recall of Rey-Osterrieth complex figure, *BNT* Boston naming test, *VFT* Verbal fluency tasks, *SDMT* Symbol digit modality test, *TMT* Trail making test, *Stroop* Stroop color word interference test

### Data analysis

Statistical analysis was performed using the Statistical Package for Social Science (SPSS version 18 for windows, Baltimore). For comparisons, the Student’s T test was applied as the variables met the normal distribution, whereas the Manne Whitney test was used for the variables that did not meet the norms for using parametric statistics. Multiple linear regression was used to evaluate the effects of gender on cognitive function. A value of *P* < 0.05 was considered statistically significant.

## Results

### Demographic and clinical characteristics of participants

Demographic and clinical data of all the PD patients are summarized in Table 1. There was no significant difference between male and female patients, with respect to age, education, years of illness duration, mean UPDRS-III score, proportion of treatment, levodopa equivalent dose or disease severity. In the comparison of treated and untreated PD patients, we found much longer disease duration in the treated PD group, without any other demographic difference. The controls did not differ on age, education background or the dementia screening.

### Cognitive performance

The results of neuropsychological tests in PD patients and controls are reported in Table 3. PD patients and controls did not differ on age, education background or the dementia screening (MMSE, *p* = 0.71). It is worth mentioning that under medication-naïve condition, male patients scored significantly worse in the MMSE (male-MMSE 28.43 ± 0.65, female-MMSE 29.20 ± 1.15, *p* = 0.014). The comparison between these two groups on specific cognitive measures revealed some differences. Three out of five domains were involved: the visual spatial function, language and attention/executive function. Specifically, PD patients attained significantly lower scores in AVLT-sum 1 to 5 (verbal memory, *p* = 0.000), Clock Drawing Test (Visual spatial function, *p* = 0.004), Verbal Fluency-City (Language, *p* = 0.000), Verbal Fluency-Alternative (Language, *p* = 0.003), Symbol Digit Modality Test (Attention/ executive function, *p* = 0.000) and Trail Making Test-A (Attention/ executive function, p = 0.000). Comparing with untreated PD, those with anti-Parkinsonism medication exhibited worse performance in Rey-copy score, Clock Drawing Test and Verbal Fluency-City. In the treated PD group, decreased score in Rey-copy test was observed, while there was no significant difference in the untreated group comparing with the controls (Table 4). Except for that, both treated and untreated PD patients displayed the same distinction in cognition tests with total PD patients as described above.

**Table 3:** Cognitive performance of patients and control (Mean ± SD)

	PD(*n* = 60)	Control(*n* = 100)	Significance
Age	59.05 ± 9.55	58.2 ± 7.57	*p* = 0.412
Education	12.86 ± 2.97	12.63 ± 3.24	*p* = 0.665
MMSE	28.98 ± 1.07	29.03 ± 0.69	*p* = 0.71
Verbal memory			
AVLT-short delayed free recall	4.91 ± 1.93	5.29 ± 2.5	*p* = 0.234
AVLT-long delayed free recall	4.47 ± 1.66	4.72 ± 2.72	*p* = 0.20
AVLT-long delayed cued recall	4.57 ± 2.06	4.69 ± 2.57	*p* = 0.819
AVLT-sum 1 to 5	15.53 ± 5.12	26.17 ± 8.15	*p* = 0.000
AVLT-recognition	20.02 ± 3.47	20.35 ± 2.89	*p* = 0.53
Non-verbal memory			
Rey-delayed recall	15.6 ± 7.58	15.16 ± 6.5	*p* = 0.74
Visuospatial function			
Rey-copy (time)	178.42 ± 61.11	165.57 ± 68.41	*p* = 0.24
Rey-copy (score)	32.98 ± 3.88	34.16 ± 1.74	*p* = .944
CDT	22.72 ± 6.05	25.81 ± 6.3	*p* = 0.004*
Language			
VFT (animals)	16.1 ± 3.6	19.43 ± 19.22	*p* = 0.19
VFT (cities)	13.76 ± 5.35	17.39 ± 5.82	*p* = 0.00*
VFT (alternative)	14.46 ± 5.2	17.11 ± 5.02	*p* = 0.003*
BNT	23.03 ± 3.66	23.63 ± 3.81	*p* = 0.34
Attention/executive function			
SDMT	31.88 ± 11.91	43.61 ± 11.6	p = 0.00*
Stroop (time)	77.95 ± 26.27	75.98 ± 24.22	*p* = 0.63
Stroop (score)	46.66 ± 4.61	46.58 ± 7.86	*p* = 0.94
TMT-A	71.37 ± 33.5	55.77 ± 24.8	*p* = 0.00*
TMT-B	164.61 ± 59.16	150.55 ± 74.35	*p* = 0.23

Note. *MMSE* Mini-mental state examination, *AVLT* Auditory verbal learning test, *Rey-delayed recall* Delayed recall of Rey-Osterrieth complex figure, *Rey-copy* Copy of Rey-Osterrieth complex figure, *CDT* Clock drawing test, *VFT* Verbal fluency tasks, *BNT* Boston naming test, *SDMT* Symbol digit modality test, *Stroop* Stroop color word interference test, *TMT* Trail making test

*p < 0.05

**Table 4:** Cognitive Performance of PD patients with and without treatment

	Treated PD (*n* = 31)	Untreated PD (*n* = 29)	Controls (*n* = 100)	*P*-value^a^	*P*-value^b^	*P*-value^c^
Age	57.03 ± 10.19	61.21 ± 8.46	58.2 ± 7.57	0.591	0.061	0.111
Gender(male)	15/31	15/29	38/100	0.402	0.204	1.000
Disease duration	6.16 ± 6.36	2.13 ± 2.79				0.000*
Education	12.70 ± 3.12	13.04 ± 2.85	12.63 ± 3.24	0.765	0.512	0.774
MMSE	29.13 ± 1.12	28.83 ± 1.00	29.03 ± 0.69	0.137	0.350	0.135
Verbal memory						
AVLT-short delayed free recall	5.04 ± 1.99	4.79 ± 1.90	5.29 ± 2.5	0.439	0.264	0.734
AVLT-long delayed free recall	4.55 ± 1.84	4.38 ± 1.47	4.72 ± 2.72	0.699	0.502	0.994
AVLT-sum 1 to 5	15.10 ± 5.83	15.96 ± 4.33	26.17 ± 8.15	0.000*	0.000*	0.378
AVLT-recognition	20.27 ± 3.95	19.78 ± 3.00	20.35 ± 2.89	0.585	0.355	0.788
Non-verbal memory						
Rey-delayed recall	13.86 ± 6.15	17.63 ± 8.67	15.16 ± 6.5	0.347	0.206	0.102
Visuospatial function						
Rey-copy (time)	175.21 ± 58.78	181.75 ± 64.34	165.57 ± 68.41	0.268	0.162	0.854
Rey-copy (score)	31.62 ± 4.82	34.44 ± 1.58	34.16 ± 1.74	0.006*	0.503	0.016*
CDT	20.41 ± 7.15	25.04 ± 3.52	25.81 ± 6.3	0.000*	0.252	0.004*
Language						
VFT (animals)	16.17 ± 3.23	16.03 ± 4.01	19.43 ± 19.22	0.358	0.193	0.632
VFT (cities)	15.10 ± 5.42	12.38 ± 5.00	17.39 ± 5.82	0.021*	0.000*	0.012*
VFT (alternative)	14.33 ± 4.01	14.59 ± 6.27	17.11 ± 5.02	0.009*	0.003*	0.715
BNT	23.10 ± 3.41	22.96 ± 3.98	23.63 ± 3.81	0.337	0.323	1.000
Attention/executive function						
SDMT	31.14 ± 13.92	32.62 ± 9.70	43.61 ± 11.6	0.000*	0.000*	0.602
Stroop (time)	76.17 ± 29.36	79.72 ± 23.15	75.98 ± 24.22	0.699	0.339	0.323
Stroop (score)	46.79 ± 4.56	46.52 ± 4.73	46.58 ± 7.86	0.027*	0.088	0.827
TMT-A	75.21 ± 44.69	67.66 ± 18.71	55.77 ± 24.8	0.004*	0.001*	0.873
TMT-B	163.96 ± 66.04	165.21 ± 53.15	150.55 ± 74.35	0.278	0.063	0.762

Note*. MMSE* Mini-mental state examination, *AVLT* Auditory verbal learning test, *Rey-delayed recall* Delayed recall of Rey-Osterrieth complex figure, *Rey-copy* Copy of Rey-Osterrieth complex figure, *CDT* Clock drawing test, *VFT* Verbal fluency tasks, *BNT* Boston naming test, *SDMT* Symbol digit modality test, *Stroop* Stroop color word interference test, *TMT* Trail making test

^a^Comparison between Treated PD and Control

^b^Comparison between Untreated PD and Control

^c^Comparison between Treated PD and Untreated PD

*p < 0.05

Table 5 summarizes the results of analyses of each gender, respectively. When compared with control, both male and female patients showed worse performance in Auditory Verbal Learning Test-sum (AVLT) 1 to 5 (Verbal memory, *p* = 0.000 for both male and female patients) and Symbol Digit Modality Test (Attention/ executive function, p = 0.000 for both male and female patients). Specifically, male patients performed worse on Verbal Fluency Test-Animals (Language, p = 0.000), Verbal Fluency-Cities (Language, *p* = 0.000) and Verbal Fluency-Alternatives (Language, *p* = 0.001), while female patients attained worse scores on Clock Drawing Test (Visual spatial function, *p* = 0.019), Boston Naming Test (Language, *p* = 0.02) and Trail Making Test-A (Attention/ executive function, *p* = 0.003).

**Table 5:** Comparison of cognitive performance between PD Patients and control by gender respectively (Mean ± SD)

	Male PD (n = 30)	Male Control (*n* = 38)	Female PD (*n* = 30)	Female Control (*n* = 62)	*P*-value^a^	*P*-value^b^	*P*-value^c^	*P*-value^d^
MMSE	28.97 ± 0.81	28.97 ± 0.79	29 ± 1.29	29.06 ± 0.62	*p* = 0.97	*p* = 0.97	*p* = 0.705	*p* = 0.54
Verbal memory								
AVLT-short delayed free recall	4.55 ± 1.68	4.74 ± 2.48	5.29 ± 2.12	5.63 ± 2.46	*p* = 0.92	*p* = 0.53	*p* = 0.153	*p* = 0.083
AVLT-long delayed free recall	4.14 ± 1.51	4.29 ± 2.56	4.79 ± 1.76	4.98 ± 2.8	*p* = 0.99	*p* = 0.99	*p* = 0.133	*p* = 0.216
AVLT-long delayed cued recall	3.96 ± 1.56	4.11 ± 2.33	5.13 ± 2.32	5.07 ± 2.66	p = 0.99	*p* = 0.91	*p* = 0.031*	*p* = 0.075
AVLT-sum 1 to 5	15.07 ± 4.32	24.37 ± 8.67	15.97 ± 5.84	27.27 ± 7.67	*p* = 0.000*	*p* = 0.000*	*p* = 0.506	*p* = 0.083
AVLT-recognition	20.52 ± 4.01	19.68 ± 2.04	19.5 ± 2.79	20.77 ± 3.26	*p* = 0.287	*p* = 0.09	*p* = 0.29	*p* = 0.069
Non-verbal memory								
Rey-delayed recall	17.16 ± 7.14	15.13 ± 7.08	14.15 ± 7.82	15.17 ± 6.18	*p* = 0.27	*p* = 0.51	*p* = 0.154	*p* = 0.978
Visuospatial function								
Rey-copy (time)	178.07 ± 67.9	159.35 ± 66.09	178.73 ± 55.47	169.4 ± 70.08	*p* = 0.27	*p* = 0.53	*p* = 0.968	*p* = 0.485
Rey-copy(score)	33.08 ± 4.13	34.11 ± 1.75	32.9 ± 3.72	34.19 ± 1.74	*p* = 0.49	*p* = 0.49	*p* = 0.867	*p* = 0.807
CDT	23 ± 4.29	25.82 ± 9.05	22.5 ± 7.22	25.8 ± 3.83	*p* = 0.16	*p* = 0.019*	*p* = 0.766	*p* = 0.989
Language								
VFT (animals)	16.45 ± 3.39	18.03 ± 5.42	15.77 ± 3.83	20.6 ± 25.66	*p* = 0.48	*p* = 0.31	*p* = 0.472	*p* = 0.556
VFT (cities)	13.9 ± 4.75	19.26 ± 5.77	13.63 ± 5.96	15.8 ± 5.43	*p* = 0.000*	*p* = 0.12	*p* = 0.852	*p* = 0.01*
VFT (alternatives)	14 ± 4.34	18.34 ± 5.41	14.9 ± 5.95	16.05 ± 4.46	p = 0.001*	*p* = 0.36	*p* = 0.511	*p* = 0.046*
BNT	24.45 ± 2.93	24 ± 4.65	21.62 ± 3.81	23.4 ± 3.21	*p* = 0.65	p = 0.02*	*p* = 0.003*	*p* = 0.45
Attention/executive function								
SDMT	31.97 ± 12.25	43.33 ± 12.24	31.79 ± 11.78	43.84 ± 11.17	*p* = 0.000*	*p* = 0.000*	*p* = 0.957	*p* = 0.849
Stroop (time)	83.43 ± 31.6	76.68 ± 18.87	72.83 ± 19.24	75.56 ± 27.05	*p* = 0.29	*p* = 0.62	*p* = 0.126	*p* = 0.827
Stroop (score)	46.04 ± 5.32	47.35 ± 12.15	47.23 ± 3.83	46.11 ± 3.42	*p* = 0.60	*p* = 0.16	*p* = 0.327	*p* = 0.451
TMT-A	64.1 ± 21.64	54.11 ± 22.81	78.89 ± 42.3	56.8 ± 26.09	*p* = 0.074	*p* = 0.003*	*p* = 0.101	*p* = 0.601
TMT-B	164.79 ± 69	152.84 ± 55.07	164.41 ± 47.71	149.11 ± 84.58	*p* = 0.43	*p* = 0.38	*p* = 0.646	*p* = 0.81

Note*. MMSE* Mini-mental state examination, *AVLT* Auditory verbal learning test, *Rey-delayed recall* Delayed recall of Rey-Osterrieth complex figure, *Rey-copy* Copy of Rey-Osterrieth complex figure, *CDT* Clock drawing test, *VFT* Verbal fluency tasks, *BNT* Boston naming test, *SDMT* Symbol digit modality test, *Stroop* Stroop color word interference test, *TMT* Trail making test

^a^Comparison between Male PD and Male Control

^b^Comparison between Female PD and Female Control

^c^Comparison between Male PD and Female PD

^d^Comparison between Male Control and Female Control

*p < 0.05

Between male and female participants, the comparison of cognitive performance is also reported in Table 5. In the control group, males performed better on Verbal Fluency-City (Language, *p* = 0.01) and Verbal Fluency-Alternative (Language, *p* = 0.046). In the PD patient group, although male and female patients did not differ on the dementia screening test, male patients performed worse on AVLT-long delayed cued recall test (Verbal memory, *p* = 0.031) and BNT test (*p* = 0.003).

We have conducted multiple linear regression using age, gender, educational level, UPDRS-III and BECK as independent variables, eliminating the cofounders of age, educational level, UPDRS and BECK (Table 6). Considering the purpose of our article, we only demonstrated the β value and *P* value of gender. After adjustment of other cofounding factors, gender difference had significant effects on the AVLT-long delayed cued recall and BNT test, consistent with the results of student T test and Manne Whitney test.

**Table 6:** Effects of gender on cognitive tests based on multiple linear regression

	Adjusted R^2^	*P*	Standardized β(gender)	*P* (gender)
MMSE	−0.027	0.587	−0.033	0.830
Verbal memory				
AVLT-short delayed free recall	0.005	0.403	0.099	0.511
AVLT-long delayed free recall	0.118	0.070	0.129	0.361
AVLT-long delayed cued recall	0.127	0.065	0.331	0.025*
AVLT-sum 1 to 5	0.044	0.235	0.037	0.799
AVLT-recognition	0.007	0.399	−0.074	0.634
Non-verbal memory				
Rey-delayed recall	0.143	0.059	−0.215	0.148
Visuospatial function				
Rey-copy (time)	0.194	0.017	−0.123	0.365
Rey-copy(score)	−0.084	0.900	−0.091	0.567
CDT	0.175	0.058	0.155	0.341
Language				
VFT (animals)	−0.042	0.680	−0.133	0.386
VFT (cities)	0.040	0.250	−0.101	0.490
VFT (alternatives)	−0.108	0.992	0.029	0.856
BNT	0.175	0.025	−0.386	0.007*
Attention/executive function				
SDMT	0.053	0.212	−0.011	0.938
Stroop (time)	0.091	0.113	−0.203	0.159
Stroop (score)	0.079	0.136	0.070	0.625
TMT-A	0.337	0.001	0.173	0.163
TMT-B	0.192	0.019	0.057	0.675

More supporting data could be accessed through emails with the corresponding authors.

## Discussion

PD patients frequently encounter neuropsychological problems. The present study has confirmed the previously reported cognitive impairment in cognitive domains including attention/executive function, visuospatial function, verbal memory and language. These aspects of cognition were all affected by the disease to varying degrees.

Dysexecutive syndrome is the most prominent prototype of early cognitive impairment in PD [22]. Deficits in this domain could be sensitively detected by measures of SDMT, which showed abnormality in this study. Our result was also in accordance with previous studies documenting visuospatial impairments by evidence of poor performance of PD patients in CDT [23]. Poor performance on free recall tasks but near normal performance on recognition and cued recall tasks in our study concur with the hypothesis that verbal memory impairment in PD has been manifested as retrieval difficulty more than encoding problems [22]. Although the majority of studies showed that language remain relatively intact in PD, we found it was impaired compared to the control group in the verbal fluency test. Verbal fluency combines the ability to retrieve the correct information and suppress the incorrect response. According to O’Brien’s report, dysfunction of various domains does not occur in isolation, but presents in association with each other [24]. In fact, though impairment in substantia nigra is most pronounced in PD, areas affected by the disease are widespread, including ventral tegmental area, dorsal raphe nucleus, hypothalamus, thalamus, hippocampus, cerebral cortex, the temporal, frontal, anterior cingulate and insular cortices [25]. Thus, it is not surprising that the cognitive deficits are due to cortical pathology and subcortical circuitry dysfunction as a whole and deficits in language may be the result of deterioration of the other cognitive functions as a whole. In addition to that, PD patients with anti-parkinsonism medication had extra deficits in Rey-copy score and stroop3 test score, which may due to the longer disease duration (*p* = 0.000) or medical effects. These results displayed a possible vulnerability of PD patients to the effects of disease duration and medication on Rey-copy score and stroop3 test.

In regard to gender differences in PD, epidemiological survey showed that the ratio of men and women who had the disease is approximately 2:1, suggesting a biological diversity [18]. However, not many studies considered gender when examining cognition in PD patients. There were some studies investigated the gender differences with a remarkable number of participants, but the lack of control groups did not permit to determine if these differences could be specific to PD patients [26, 27]. The present study highlights the role of gender differences associated with cognitive functions. The conclusion was strengthened by the study design of age and education matched control groups. Our study showed a disparity between male and female patients in two domains of cognition. Male patients surpassed female patients on BNT, a measure less commonly used to assess frontal lobe dysfunction [28], while female patients were superior on verbal retrieval test, reflecting the impairment of hippocampus [29]. Since no significant differences were observed in these two measures between male and female controls, it is reasonable to infer that gender-based differences existed in PD patients. In a cross-sectional study of the effect of gender on BNT which recruited 1111 healthy elderly subjects, there was also a tendentiously while non-significantly higher score of males (*p* = 0.08) [30]. Meanwhile, it reveals that age and educational level had more powerful effect on BNT. On the other hand, other studies found no effect of gender on the BNT. Therefore, there was possibility that the gender difference in BNT was due to the natural difference between men and women, which was unrelated to PD.

One consideration centers on our results is the role estrogen plays in the pathogenesis of PD. Even though the menopausal condition at experiment might be heterogeneous of the female PD patients in our cohort, the lifetime cumulative level of estrogen also played an important part in the pathogenesis of Parkinson’s disease [31, 32]. According to other’s reports, it is still a mystery as to the mechanism of estrogen acting on the dopaminergic system [33, 34]. In addition, the changes in other neurotransmitter systems, such as cholinergic, noradrenergic, serotonergic, also contribute to the multiple neuropsychological impairments. The interactions of these neurotransmitter systems make the role of estrogen even more complex. Another frequently-used theory to explain the gender differences is “cognitive reserve”, which posits that premorbid condition may generate distinctions in clinical presentation [35–37]. Although the subjects in our study have been adjusted for education, it may not be sufficient to rule out the impact of other socioeconomic factors such as occupation, income and social status. Thus better performance in verbal memory by female patients may indicate a larger cognitive reserve in this aspect. Likewise, advantages in naming by male patients may suggest a later onset of impairment and greater reserve of prefrontal function than female counterpart. The gender differences might also be associated with neural organization [38]. Some studies shown that greater bihemispheric representation were more prominent in women taking verbal memory task and men taking visuospatial task [39, 40]. Others found right hemispheric lateralization for males and bilateralization for females [41, 42]. Though no consensus has been reached, differences in neural lateralization render certain aspects of cognition more sensitive to neuropathological changes in a gender-specific manner, which lead to dissimilar manifestation in male and female patients.

When interpreting these data, several limitations should be acknowledged. First of all, the relatively small sample size may limit the generalization of these data. As several studies pointed out, normal elderly women performed better in tests involving verbal components [37, 43].Thus, whether female PD patients’ better performance in RVLT-long delayed cued recall was due to the influence of the disease needs further study with larger sample size. Secondly, as Cronin-Golomb described, side of disease onset may also influence the cognition of the patients [44]. Should this be the case, more detailed division of participants by both gender and side of disease onset would provide stronger evidence. Finally, another limitation is the influence of medication. Though anticholinergic medications were ruled out and levodopa equivalent dose were well matched between groups, the underlying effect of dopaminergic medication may still change the natural pathological development of neurodegeneration.

In conclusion, our study indicates that cognitive impairment was common in PD patients even in the absence of dementia. PD patients who underwent anti-parkinsonian treatment had worse cognitive impairment than untreated ones. In light of the above mentioned observations, we hypothesized that genders may have a different presentation of cognitive impairment in PD patients. Sex influences on brain anatomy, chemistry and functions are poorly understood. Increased knowledge on possible gender effects in PD would provide an enhanced insight in underlying pathological mechanisms, and has potential implications for the diagnosis and treatment of PD.
